# Regulation of type 3 fimbria expression by RstA affects biofilm formation and virulence in *Klebsiella pneumoniae* ATCC43816

**DOI:** 10.1128/spectrum.03076-24

**Published:** 2025-05-15

**Authors:** Xiaoyun Zhang, Yaxuan Zhai, Jie Zhu, Zhichen Zhu, Yicheng Wen, Qizhao Gao, Liang Wang, Jiayao Lin, Yan Qian, Liang Chen, Hong Du

**Affiliations:** 1 Department of Clinical Laboratory, The Second Affiliated Hospital of Soochow University 105860, Suzhou, Jiangsu, China; 2 Department of Clinical Laboratory, The Affiliated Huaian No.1 People's Hospital of Nanjing Medical University 91596, Huai'an, Jiangsu, China; 3 Department of Pharmacy Practice, School of Pharmacy and Pharmaceutical Sciences, University at Buffalo 15497, Buffalo, New York, USA; The Pennsylvania State University, University Park, Pennsylvania, USA

**Keywords:** *Klebsiella pneumoniae*, RstA, type 3 fimbriae, biofilm formation, virulence

## Abstract

*Klebsiella pneumoniae* causes both community-acquired and healthcare-associated infections, presenting a major therapeutic challenge to global public health. RstBA is a common two-component regulatory system that controls downstream gene expression in certain *Enterobacteriaceae* species. However, the role of RstBA in *K. pneumoniae* infection remains unknown. To determine its function, a wild-type *K. pneumoniae* strain (ATCC43816) and *rstA* mutant and complementation strains were constructed. Phenotypic experiments and *in vivo* animal infection assays demonstrated that deletion of *rstA* decreased virulence and biofilm formation. RNA sequencing analysis of ATCC43816 and *rstA* mutant strains was performed to study the regulatory mechanisms, revealing differential expression of genes involved in arginine and proline metabolism, phenylalanine metabolism, and quorum sensing. In addition, the *mrkI* and the *mrkABCDF* gene cluster, which regulates and encodes type 3 fimbriae, exhibited lower expression in the absence of *rstA*, possibly related to decreased virulence and biofilm formation. Quantitative real-time reverse transcription PCR, promoter activity assays, and electrophoretic mobility shift assays were conducted to identify the transcriptional regulation of *mrkI* and *mrkABCDF* by *rstA*. Our findings show that *rstA* regulates type 3 fimbriae expression by regulating *mrkI* indirectly and regulating *mrkA* directly by binding to its promoter. This study provides new insights into the functional importance of RstA in regulating biofilm formation and virulence in *K. pneumoniae*.

**IMPORTANCE:**

*Klebsiella pneumoniae* is an opportunistic pathogen that has become a significant cause of community-acquired and nosocomial infections. The rise of hypervirulent and multi-drug-resistant *K. pneumoniae* poses a significant threat to public health. The two-component regulatory system is a typical signal-sensing and stress-response system widely distributed in bacteria, playing a critical regulatory role in bacterial infection. Through *in vivo* and *in vitro* experiments, we demonstrate that *rstA* regulates the expression of type 3 fimbriae by regulating *mrkI* indirectly and *mrkA* directly, thereby playing an essential role in the virulence and biofilm formation of *K. pneumoniae*. Understanding the regulatory mechanism of RstA in *K. pneumoniae* provides a proof-of-concept for identifying new genetic targets for controlling *K. pneumoniae* infection, which may aid in the development of therapeutic drugs.

## INTRODUCTION

*Klebsiella pneumoniae* is an opportunistic gram-negative pathogen that causes a range of infections, including pneumonia, urinary tract infections, bacteremia, meningitis, endophthalmitis, and pyogenic liver abscesses, posing a substantial threat to public health (1, 2). Since hypervirulent *K. pneumoniae* (hvKP) was first reported in Taiwan Province in the 1980s, it has emerged as another prevalent strain besides classical *K. pneumoniae* (3). The hvKP strain can disseminate to distant sites, such as the lungs, liver, kidneys, spleen, fascia, eyes, and the central nervous system, causing suppurative liver abscesses and other invasive syndromes. Owing to the rapid progression of the infection, patients with hvKP infections usually have a poor prognosis (4, 5). Multiple virulence factors are associated with *K. pneumoniae* infections, such as capsular polysaccharides, lipopolysaccharides, fimbriae, and iron acquisition systems (6, 7). Fimbriae are also key virulence factors, with *K. pneumoniae* typically expressing two well-characterized types: type 1 and type 3. These fimbriae mediate bacterial adherence and the invasion of host cells, which is crucial for developing infections (8). Type 3 fimbriae are the main determinants of biofilm formation in *K. pneumoniae*. Biofilms provide protection and resistance against invasive factors such as host immune response, antibacterial agents, disinfectants, and extreme environmental factors (such as ultraviolet radiation, extreme pH, extreme temperature, high salinity, and high pressure), which are key factors in the occurrence of nosocomial infections (9–11). Type 3 fimbriae are encoded by the *mrkABCDF* operon, which shares the *mrkA* gene promoter (12, 13). Their expression is influenced by secondary messengers such as cyclic di-GMP, *mrkH*, *mrkI*, *mrkK, mrkJ*, histone-like nucleic acid structure protein, and *fur* (14–16). However, the specific regulatory mechanisms governing type 3 fimbriae in *K. pneumoniae* remain unclear. Understanding these mechanisms is essential for the control of infections.

*K. pneumoniae* senses various environmental signals, including temperature, pH, nutrient restriction, oxygen availability, and osmotic pressure, to successfully infect its host (17, 18). Bacterial two-component regulatory systems (TCSs) are essential for forming an effective supervisory network (19). A TCS typically comprises a sensor histidine kinase and cytoplasmic response regulators. The histidine kinase monitors environmental signals, self-phosphorylates its own conserved histidine residue, and transfers the phosphorylated group to a specific aspartate residue of a cognate response regulator, which regulates the transcription of target genes (20). The RstBA system is a typical TCS consisting of the reactive histidine kinase RstB and its cognate regulator RstA (21). RstBA has been implicated in regulating virulence, adhesion, nitrogen metabolism, acid adaptation, biofilm formation, motility, and iron acquisition in *Vibrio alginolyticus*, *Photobacterium damselae subsp. damselae*, enterohemorrhagic *Escherichia coli* O157 (EHEC O157), *Salmonella enterica*, and avian pathogenic *E. coli* (APEC) (22–28). However, the exact mechanism by which RstA regulates the adaptation of *K. pneumoniae*, particularly its role in virulence regulation, remains unclear.

In this study, we aimed to elucidate the role of RstA in hvKP ATCC43816. By constructing *rstA* mutant and complementation strains and employing RNA sequencing, we identified downstream transcriptional regulatory mechanisms governing type 3 fimbriae expression. Our findings contribute to a deeper understanding of the molecular mechanisms underlying *K. pneumoniae* pathogenesis and may inform the development of targeted therapeutic strategies to combat infections caused by this pathogen.

## MATERIALS AND METHODS

### Bacterial strains, plasmids, primers, and culture conditions

The bacterial strains, plasmids, and primers used or constructed in this study are listed in Tables 1 and 2. The strains were cultured in Luria-Bertani (LB) medium. The antibiotics used include kanamycin (50 µg/mL), rifampicin (100 µg/mL), apramycin (50 µg/mL), and gentamicin (50 µg/mL).

**TABLE 1:** Bacteria and plasmids used in the study^*a*^

Strain or plasmid	Description	Source
*K. pneumoniae* strains		
ATCC43816	K2 serotype	Laboratory stock
Kp: *ΔrstA*	ATCC43816 with deletion of *rstA*	This study
Kp: *ΔrstA/cprstA*	Kp: *ΔrstA* complemented with *rstA*	This study
Kp: *ΔrstA/cpET28a*	Kp: *ΔrstA* complemented with pET28a-rpsl	This study
*E. coli* strains		
DH5a	Cloning host	Laboratory stock
BL21	Express protein	Laboratory stock
Plasmids		
pET28a-rpsl	Km^r^, cloning vector	Laboratory stock
pET28a-rpsl-rstA+	Km^r^, cloning vector containing *rstA*	This study
pHRP309-*lacZ*	GN^r^	Laboratory stock
pET-28a	Km^r^, protein expression vector	Laboratory stock
pET-28a-*rstA*+	Km^r^, pET-28a containing *rstA*	This study
pSGKP	Rif^r^	Laboratory stock
pCasKP	Apr^r^	Laboratory stock

^*a*^ Km^r^, kanamycin resistance; Rif^r^, rifampicin; Apr^r^, apramycin; GN^r^, gentamycin.

**TABLE 2:** Primers used in this study

Target	Primer (5'−3')
Gene deletion	
*rstA*-N20-F	CCCAAGCTTCCACAGCAGATCGAAATCGGGTTTTAGAGCTAGAAATAGCAAGTT
*rstA*-N20-R	CTAGTCTAGAGGATCCCCCGGGCTGCAG
*rstA*-HA1	CCGGTTTCAAAGTGATGC
*rstA*-HA2	ATGTGCAATAGACACTAAGCAGCCCTTCTG
*rstA*-HA3	CAGAAGGGCTGCTTAGTGTCTATTGCACATCAGTAAAAGAAG
*rstA*-HA4	CTGCGCTAGTGGAAACG
Gene complementation	
c-*rstA*-F	CCGGAATTCATGAATAAAATCGTTTTTGTTGAGGATGACCCTGAGG
c-*rstA*-R	CCCAAGCTTTTAGTTATCCCAGGCGTGAGGGGC
RT-PCR	
*rstA*-F	GCTGATCGCCGCCTATCTCG
*rstA*-R	ACAGAGCGTCATTCCGTCCTTG
*mrkI*-F *mrkI*-R	ATTGTCGGGCTGTGCAGAGAG GCAGTTCGCTGACGCCTTTG
*mrkA*-F	CGGCAGCAGCGGATACTTACC
*mrkA*-R	GTTCACGCCCAGTTTGCTTACG
*mrkB*-F *mrkB*-R	GCCACACCGGATACCATTACCAC GATTCAGCCACCACAGCGTCTC
*mrkC*-F *mrkC*-R	GGCAGCAGCAGCAGGGTAAAG CGCCGCTGACGATGGAGTTG
*mrkD*-F *mrkD*-R	TGGCGAAAGGCATTGGCATCC GCAGCGGTATGGTGATGTAGCG
*mrkF*-F *mrkF*-R	ATGAAACCGCAGCCGTTTACCC ACCCGACGCACCTCATCCTG
*lacZ* fusion	
*mrkI*-lacZ-F *mrkI*-lacZ-R	ACGCGTCGACCGCCAAATATTCTGCTGATTCC CCGGAATTCGTTGGGTTATGATACTGGTGTTG
*mrkA-lacZ*-F	ACGCGTCGACATGGGCTGCCCTTGTTCAGG
*mrkA-lacZ*-R	GCTCTAGACGGTAACTTTACCGAAGAAATTAACCTGGC
Protein expression	
*rstA*-P-F	GGGAATTCCATATGATGAATAAAATCGTTTTTGTTGAGGATGACCC
*rstA*-P-R	CCGCTCGAGTTAGTTATCCCAGGCGTGAGG
Electrophoretic mobility shift assay (EMSA)	
*mrkI-EMSA-F mrkI-EMSA-R*	CCCTTGTAAATAGTTGTCGTGAGGCG TTCCCGCTGGGCGGTTTTAC
*mrkA*-EMSA-F	GCGTGGCAAAAAGTGCGATTT
*mrkA*-EMSA-R	TTCCTTGTCAGAGTGAATTACGAATCAATGAG
*16S*-EMSA-F	CGGTATCGCCTTTACCGGTCA
*16S-*EMSA-R	TGGGTATTAACAATCATTTTGATGGCGAG

### Antimicrobial susceptibility testing

Antimicrobial susceptibility testing was conducted utilizing a BD PhoenixTM 100 Automated Microbiology System. The findings were evaluated using the breakpoints provided by the Clinical and Laboratory Standards Institute (CLSI M100, 34th edition).

### Construction of gene mutant and complementation strains

A CRISPR/Cas9-mediated genome editing system was used to generate the ATCC43816 *rstA* mutant (29). Plasmid pCasKP was transformed into ATCC43816, and single guide RNA (sgRNA) fragments targeting the 20-nt base-pairing region (N20) of *rstA* were amplified from plasmid pSGKP. Then, the PCR products were ligated into enzymatically digested pSGKP. Co-transformation of DNA repair templates and pSGKP-*rstA*-N20 plasmids with pCasKP-positive ATCC43816 generated the *rstA* mutant. In addition, rstA was cloned into the pET28a-rpsL vector in a reverse orientation relative to the T7 promoter. The constitutive rpsL promoter was introduced upstream of the gene to drive its expression. The recombinant plasmid, pET28a-rpsL-rstA, was introduced into *E. coli* DH5α cells. Finally, *ΔrstA* carrying the recombinant plasmid pET28a-rpsL-rstA was generated as the *ΔrstA/cprstA* (complementation) mutant, and the *ΔrstA* strain harboring the empty vector pET28a-rpsL (*ΔrstA/cpET28a*) served as a control.

### Bacterial growth curves

To detect the growth rate of bacteria *in vitro*, wild type (WT), *ΔrstA*, *ΔrstA/cprstA*, and *ΔrstA/cpET28a* strains were cultured overnight in LB broth, and the bacterial solution was diluted 1:100 for subculture. The diluted solution was added to a 96-well sterile plate and incubated in a BMG LABTECH automatic microplate reader at 37°C with shaking at 250 rpm. OD_600_ bacterial density was measured every half hour.

### Quantification of biofilm amount

The WT, *ΔrstA*, *ΔrstA/cprstA*, and *ΔrstA*/*cpET28a* strains were cultured overnight in LB broth, then diluted 1:100 for subculture and grown to the late exponential phase. Next, 20 µL of the culture was transferred to a 96-well plate containing 180 µL of brain heart infusion broth and incubated at 37°C for 48 h to form a biofilm. The biofilm was washed three times with phosphate-buffered saline, fixed with formaldehyde for 20 min, and stained with 1% crystal violet solution at 37°C for 30 min. The stained biofilm was dissolved with 33% glacial acetic acid, and absorbance was measured at OD_595_ (30). The absorbance data were gained from three repeated experiments.

### Serum killing assay

The WT, *ΔrstA*, *ΔrstA/cprstA*, and *ΔrstA/cpET28a* strains were cultured overnight in LB broth, and the bacterial solutions were diluted 1:100 for subculture and grown to the late exponential phase. After dilution, 250 µL of bacteria (10^5^ CFU/mL) was incubated with 750 µL of serum for 2 h, and 100 µL was removed every hour to coat the plate. The plate was incubated at 37°C overnight for colony counting (31).

### *Galleria mellonella* killing assay

*Galleria mellonella* larvae were used to detect bacterial virulence (32, 33). The WT, *ΔrstA*, *ΔrstA/cprstA*, and *ΔrstA/cpET28a* strains were cultured in LB broth overnight and then diluted 1:100 for subculture and grown to the late exponential phase. The bacterial solution was washed twice with saline. *G. mellonella* larvae were randomly divided into groups of 20. Each larva was injected with a bacterial solution (10^5^ CFU), whereas the control group was injected with saline. Larvae unresponsive to stimuli were considered dead, and mortality was recorded every 12 h for 96 h. The experiment was repeated at least three times.

### Mouse infection assay

Bacterial virulence was evaluated through intraperitoneal injection of bacterial strains into mice (34). Five C57BL/6J mice per group were injected intraperitoneally with saline, WT, *ΔrstA*, *ΔrstA/cprstA*, and *ΔrstA/cpET28a* strains (10^3^ CFU). After 24 h, blood samples were collected from the hearts of the mice, and serum levels of interleukin (IL)−6, IL-10, and tumor necrosis factor (TNF)-α were detected using the enzyme-linked immunosorbent assay method. Liver and lung tissues from each group of mice were fixed, embedded, and stained with hematoxylin and eosin for histopathological examination. The animal experiments were approved by the Animal Experiment Ethics Committee of the Second Afﬁliated Hospital of Soochow University (ethics number JD-LK-2020-029-01).

### RNA sequencing and differential expression gene analysis

Total RNA was extracted from strains grown overnight in LB broth using FastPure Cell/Tissue Total RNA Isolation Kit V2-RC112 (Vazyme, China). RNA sequencing was performed at Novogene Bioinformatics Technology Co., Ltd. (Novogene, China). Data were analyzed using the free online Majorbio Cloud platform, and differentially expressed genes (DEGs) were identified using the DESeq2 package; *P*-value < 0.05 and | log2(fold change) | >2 indicated significance in differential expression. Kyoto Encyclopedia of Genes and Genomes (KEGG) and GO enrichment analyses were conducted using KOBAS 2.0 and Goatools to identify statistically significant differences in the functional and metabolic pathway levels.

### Quantitative reverse transcription PCR

Total RNA was extracted from strains grown overnight in LB broth using the FastPure Cell/Tissue Total RNA Isolation Kit V2-RC112 (Vazyme, China) and converted to cDNA following the manufacturer’s instructions. Gene transcription levels were detected through quantitative reverse transcription PCR (qRT-PCR) using the Taq Pro Universal SYBR qPCR Master Mix Kit (Vazyme Biotech Co., Ltd., China). Gene expression was normalized to the abundance of 16S rRNA transcripts. The relative difference in mRNA levels was calculated using the 2^*−ΔΔ*Ct^ method (35). All qRT-PCR assays were performed in triplicate.

### Promoter activity assay

The *lacZ* fusion method was used for the promoter activity assay (36). The DNA fragments of the putative promoter region of *mrkI and mrkA* were amplified and introduced into a pHRP309 plasmid, which carried a promoter-less *lacZ* reporter gene (37). The recombinant plasmid was introduced into WT and *ΔrstA* strains, and the β-galactosidase activity in the bacterial lysate was measured using a β-galactosidase enzyme assay system with reporter lysis buffer (Promega, USA).

### Construction and purification of His-RstA

The coding region of *rstA* was amplified through PCR and cloned into the NdeI/HindIII site of pET28a. The resulting plasmid, pET28a-*rstA*, was transformed into *E. coli* BL21. The recombinant protein was induced using 0.02 mM isopropyl β-D-thiogalactopyranoside at 16°C overnight. The recombinant proteins were purified from the bacterial lysate using His-Bind resin affinity chromatography. The purified proteins were then dialyzed against a buffer (50 mM Tris-HCl, 50 mM ZnCl_2_, and 50 mM NaCl) at 4°C overnight and assessed purity using SDS-PAGE.

### Electrophoretic mobility shift assay

The promoter-proximal DNA region of *mrkI* and *mrkA* was amplified through PCR. A total of 100 ng of promoter-proximal DNA fragment was incubated with purified RstA protein and binding buffer at 37°C for 30 min. Then, 10× loading buffer was added to the reaction mixture, and the sample was subjected to PAGE. The gel was stained with GelRed (Biotium, USA) for 30 min at room temperature and visualized using a UV transilluminator (38, 39).

### Statistical analysis

Differences between experimental groups were analyzed using Student’s *t*-test and one-way analysis of variance. Survival curves were evaluated using the log-rank test. Differences were considered significant at *P* < 0.05.

## RESULTS

### RstA does not affect the growth of ATCC43816

**Fig 1:**
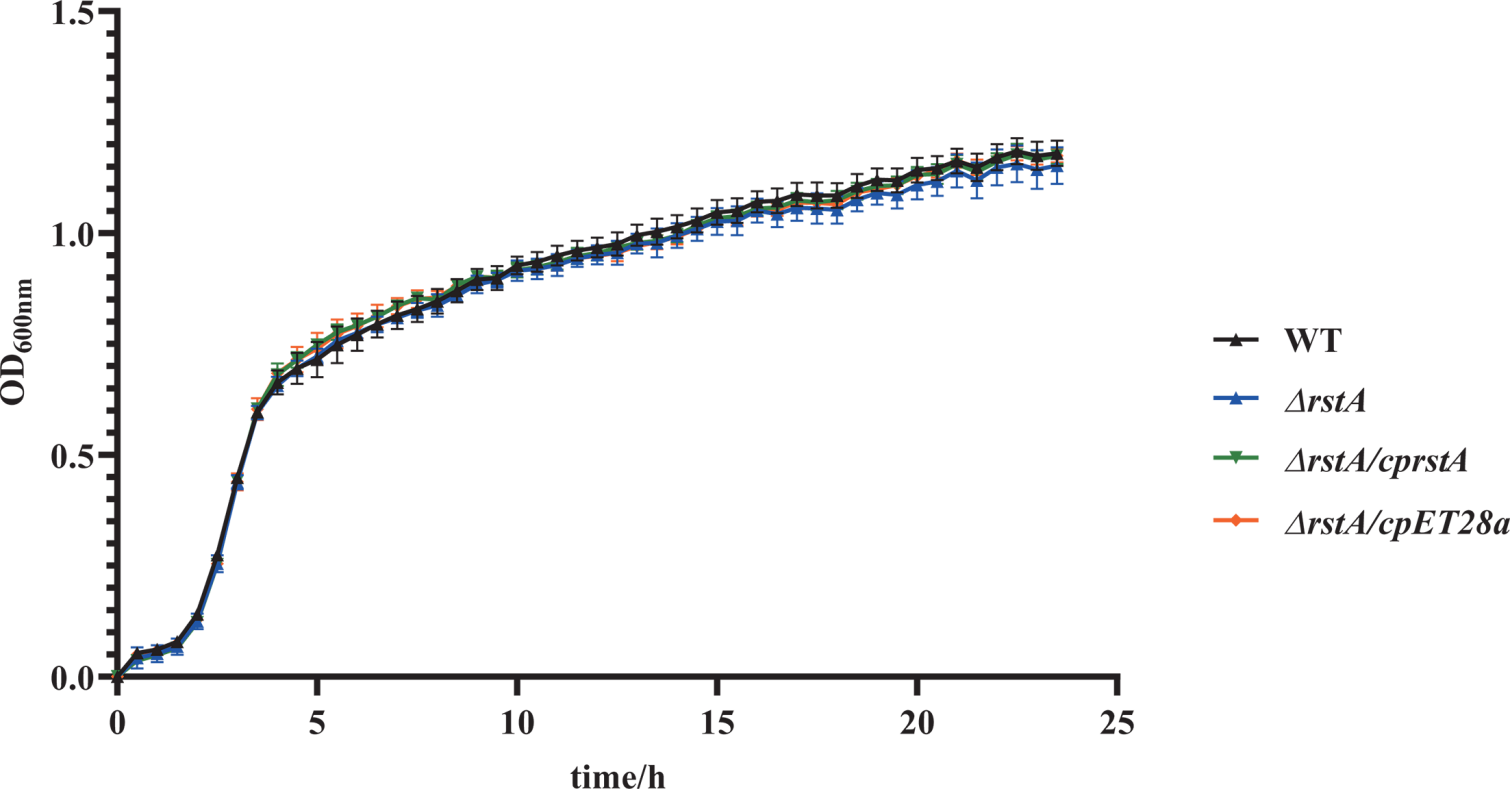
Bacterial growth curves. The growth rates of the WT, *ΔrstA*, *ΔrstA/cprstA*, and *ΔrstA/cpET28a* strains were similar, as monitored by measuring the optical density (OD_600_) at half-hour intervals over 24 h.

The *rstA* mutant and complementation strains were successfully constructed and verified using RT-PCR (Fig. S1). As shown in Fig. 1, the growth curves of the WT, *ΔrstA*, *ΔrstA/cprstA*, and ΔrstA/cpET28a strains in LB broth showed no significant difference. Antimicrobial susceptibility testing revealed that ATCC43816 and *ΔrstA* were susceptible to all the tested antibacterial agents. Comparative analysis of the minimum inhibitory concentrations for 18 antibiotics between ATCC43816 and *ΔrstA* demonstrated no significant differences (Table S1). These findings indicated that the deletion of *rstA* did not affect the growth or antimicrobial susceptibility of hvKP ATCC43816.

### RstA deletion decreases biofilm formation

**Fig 2:**
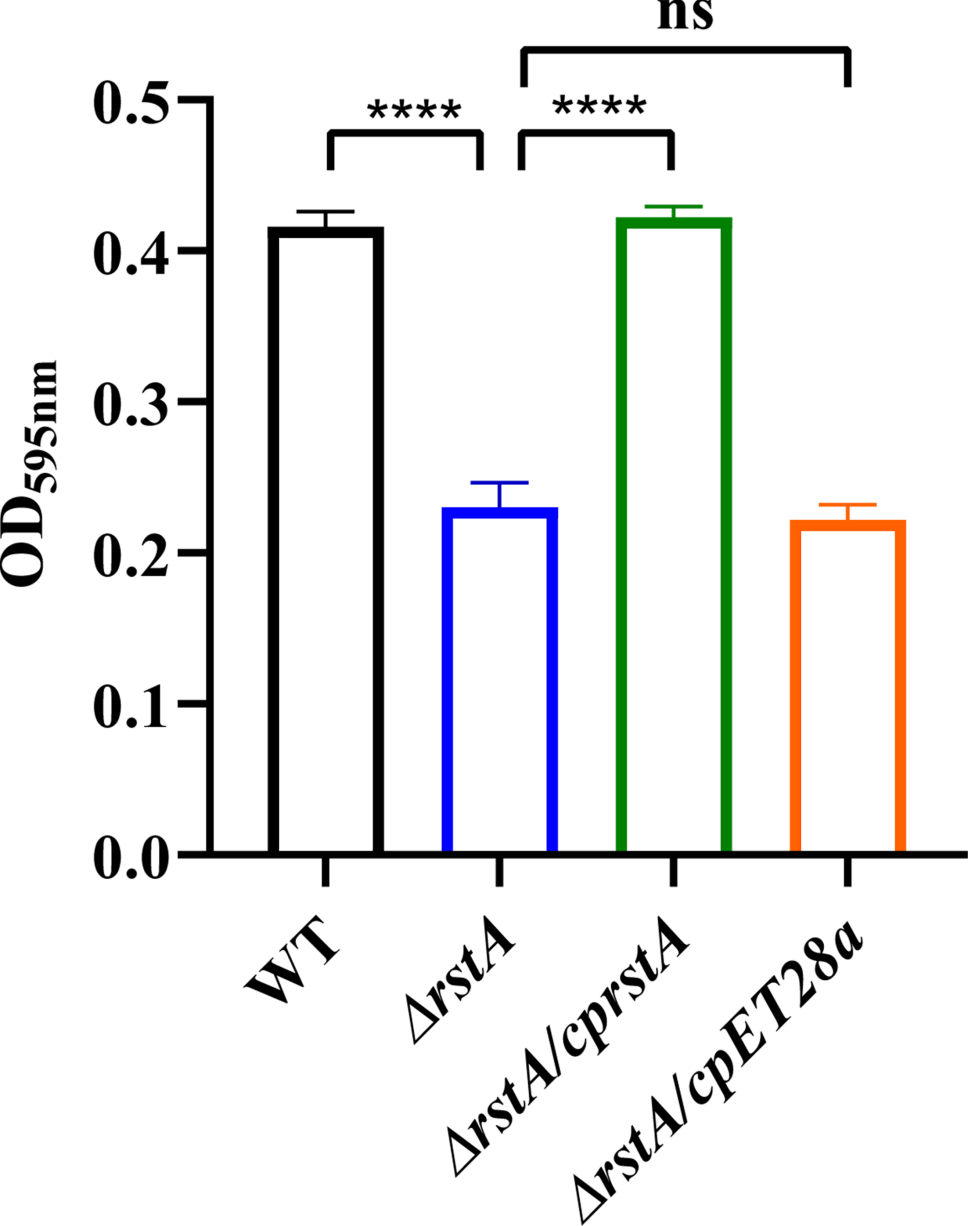
*In vitro* biofilm formation. Biofilm formation was measured by the crystal violet staining method, and the solution was measured at OD595. There were significant differences in biofilm formation among the WT, *ΔrstA*, and *ΔrstA/cprstA* strains (****, *P* < 0.0001; ns, not significant).

Biofilm formation assays (Fig. 2) demonstrated that biofilm production in the *ΔrstA* strain was significantly reduced compared to the WT strain. The complementation strain (*ΔrstA/cprstA*) restored biofilm formation to near wild-type levels, suggesting that *rstA* positively regulates biofilm formation in *K. pneumoniae*.

### RstA deletion decreases serum resistance of ATCC43816

**Fig 3:**
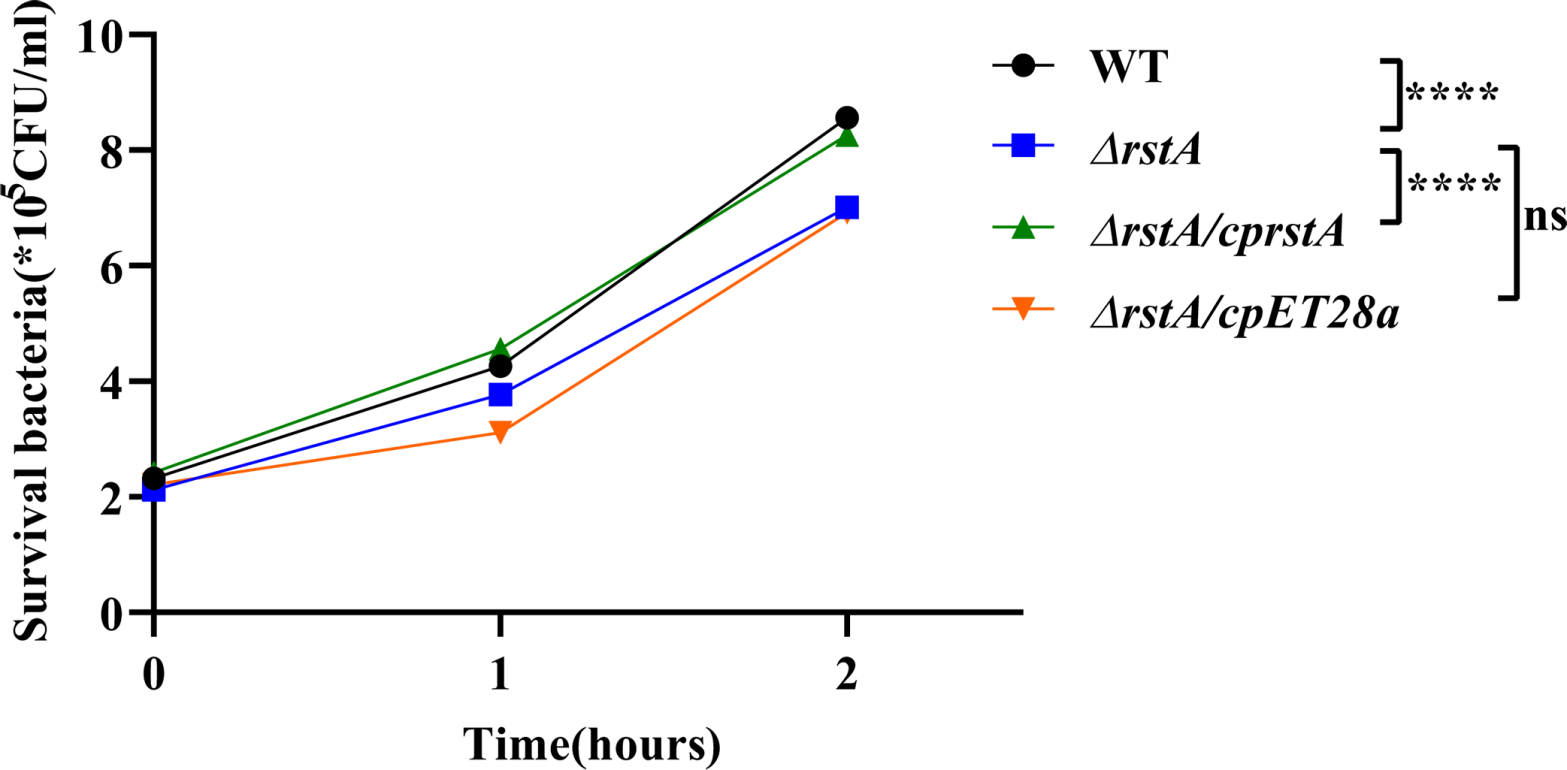
Effect of human serum on the WT, *ΔrstA*, *ΔrstA/cprstA*, and *ΔrstA/cpET28a* strains. Bacteria were mixed with serum for 2 h. The *ΔrstA* strain showed significantly lower resistance compared to the WT and *ΔrstA/cprstA* strains (****, *P* < 0.0001; ns, not significant).

The serum resistance of the WT and *ΔrstA* strains *in vitro* was studied using a serum-killing assay. As shown in Fig. 3, significant differences were observed among the WT, *ΔrstA*, and *ΔrstA/cprstA* strains. After 2 h of incubation, the mean survival count of the *ΔrstA* strain was 7.09 × 10^5^ CFU/mL, which was significantly lower than that of the WT (8.49 × 10^5^ CFU/mL) and *ΔrstA/cprstA* strains (8.36 × 10^5^ CFU/mL), indicating that *rstA* contributes to serum resistance in *K. pneumoniae in vitro* (*P* < 0.0001).

### RstA deletion decreases the virulence of ATCC43816 *in vivo*

**Fig 4:**
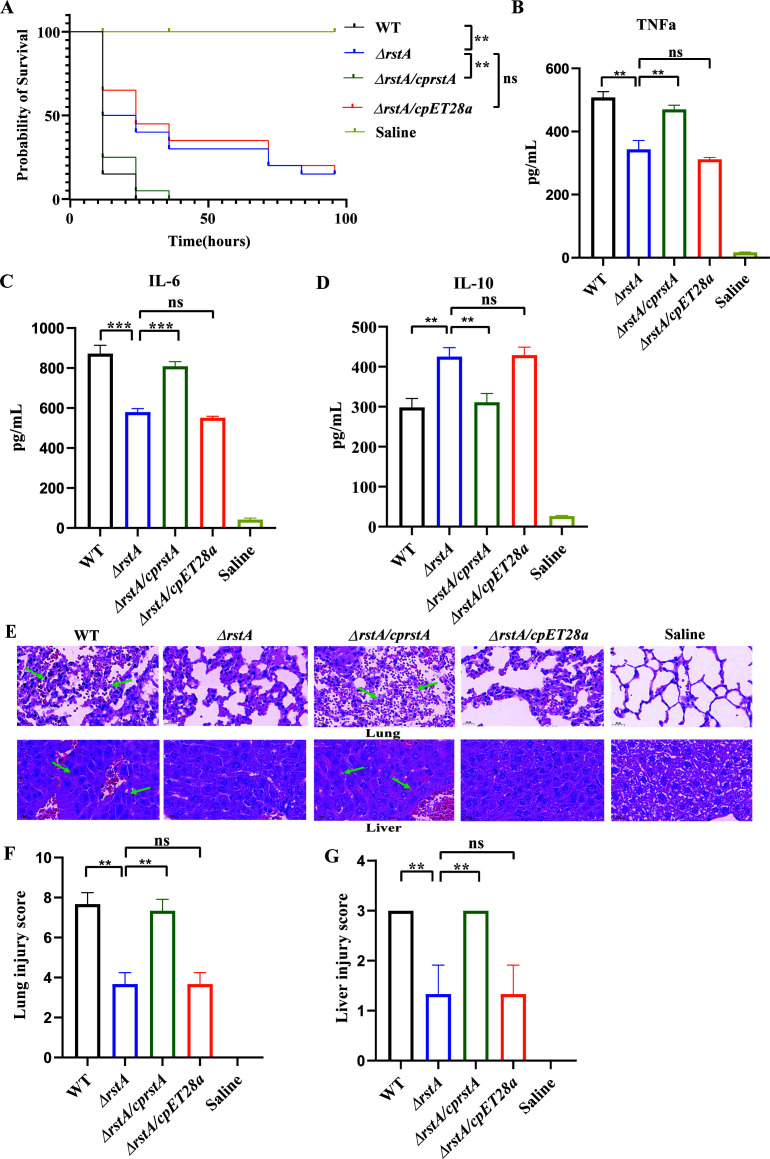
Deletion of *rstA* decreases the virulence of ATCC43816 *in vivo*. (**A**) Analysis of *G. mellonella* larva survival over 96 h following injection with 10^5^ CFU of the WT, *ΔrstA*, *ΔrstA/cprstA*, and *ΔrstA/cpET28a* strains. The survival rate of larvae infected with *ΔrstA* was significantly higher than that of larvae infected with WT and *ΔrstA/cprstA* (**P < 0.01; ns, not significant). (**B through D**) Enzyme-linked immunosorbent assay (ELISA) of inflammatory cytokines production in mouse serum. (**B**) Compared to the WT and *ΔrstA/cprstA* groups, TNF-α production in the *ΔrstA* group was significantly decreased (**, *P* < 0.01; ns, not significant). (**C**) Compared to that in the WT and *ΔrstA/cprstA* groups, IL-6 production in the *ΔrstA* group was significantly decreased (***, *P* < 0.001; ns, not significant). (**D**) Compared to that in the WT and *ΔrstA/cprstA* groups, IL-10 production was significantly increased (**, *P* < 0.01; ns, not significant). (**E**) Histopathology of mouse lungs and liver. The lungs and livers of mice were dissected and stained 24 h after injection. Lung tissues from the WT and *ΔrstA/cprstA* groups were damaged and filled with blood, whereas liver tissues contained inflammatory lesions and blood cells. The *ΔrstA* group showed mild inflammatory pathological changes in both lung and liver tissues. Arrows show blood cell or inflammatory lesions in lung and liver tissues. (**F**) Smith’s lung injury score. (**G**) Nonalcoholic atttosteatohepatitis (NAS) liver injury score.

The impact of *rstA* on the *in vivo* virulence of ATCC43816 was initially assessed using a *G. mellonella*-killing assay. Strains were injected into *G. mellonella* larvae, and the survival rate at a specific time point was used as a virulence index. As shown in Fig. 4A, after 72 h of infection, the survival rate of *ΔrstA* was about 20%. The survival rate of ATCC43816 and *ΔrstA/cprstA* was 0%, which was significantly lower than that of *ΔrstA* (*P* < 0.01), suggesting that *rstA* deletion reduces virulence in *G. mellonella* larvae.

To further verify this finding, C57BL/6J mice were infected peritoneally with WT, *ΔrstA*, *ΔrstA/cprstA*, and *ΔrstA/cpET28a* strains. Whole blood, lungs, and liver tissues were collected from mice 24 h after injection. As shown in Fig. 4B through D, mice injected with the *ΔrstA* strain had significantly lower serum levels of pro-inflammatory factors IL-6 and TNF-α and significantly higher levels of the anti-inflammatory factor IL-10 compared to the WT. Lung tissue congestion in mice injected with WT and *ΔrstA/cprstA* was evident under the microscope, and hepatocyte necrosis along with inflammatory lesions of varying sizes was visible in the liver tissue. The lung and liver histopathology images have been quantified using the Smith and NAS scores, respectively. The higher the score, the more severe the damage. The scores in mice injected with *ΔrstA* were significantly reduced compared to WT and *ΔrstA/cprstA* groups (Fig. 4E through G; Fig. S2). These results suggest that *rstA* deletion significantly reduces bacterial virulence in mice.

### RstA positively regulates *mrkABCDF* expression by regulating *mrkI* indirectly and *mrkA* directly

RNA sequencing analysis comparing ATCC43816 and *ΔrstA* strains identified 105 DEGs (including 51 upregulated and 54 downregulated genes) in *ΔrstA* (Table S2). As shown in Table 3, KEGG enrichment analysis indicated that these genes are involved in several biological processes, including arginine and proline metabolism, phenylalanine metabolism, and quorum sensing. Notably, the transcription level of gene *mrkI*, which regulates type 3 fimbriae expression, was significantly decreased in *ΔrstA*. As type 3 fimbriae are the main component of biofilm formation in *K. pneumoniae*, the decreased expression of type 3 fimbriae could lead to a decrease in biofilm formation and virulence in ATCC43816, which was consistent with the previous results.

**TABLE 3:** Expression of the genes influenced by deletion of *rstA*

Category	Gene ID	Gene name	Log_2_ fold change for *ΔrstA vs* WT	Proposed function
Arginine and proline metabolism	VK055_RS05115	VK055_RS05115	−2.24	Aspartate aminotransferase family protein
	VK055_RS23005	*speB*	3.29	Agmatinase
Phenylalanine metabolism	VK055_RS05230	*paaC*	−3.3	Phenylacetyl-CoA epoxidase subunit PaaC
	VK055_RS05215	*paaF*	−6.83	Dehydroadipyl-CoA hydratase PaaF
	VK055_RS05235	*paaB*	−2.77	Phenylacetyl-CoA epoxidase subunit PaaB
	VK055_RS01710	*dmpG*	3.01	4-Hydroxy-2-oxovalerate aldolase
Quorum sensing	VK055_RS00835	VK055_RS00835	−2.07	ATP-binding cassette domain-containing protein
	VK055_RS21190	VK055_RS21190	6.02	ABC transporter permease
	VK055_RS09800	VK055_RS09800	−3.18	ABC transporter permease
	VK055_RS21185	VK055_RS21185	−5.62	ABC transporter permease
Fimbriae expression regulation	VK055_RS21465	*mrkI*	−5.84	Type 3 fimbriae transcriptional regulator

**Fig 5:**
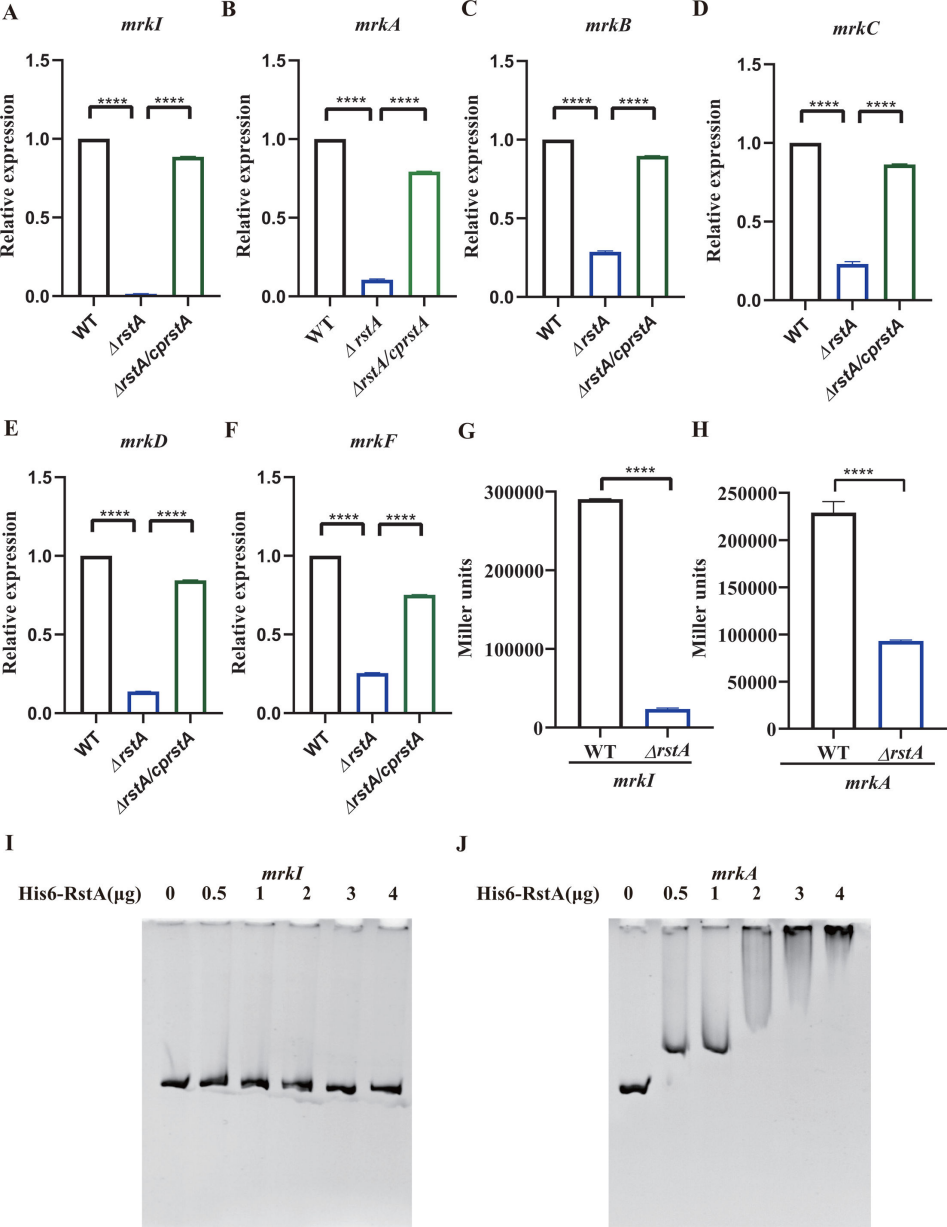
Positive regulation of *mrkI* and *mrkABCDF* gene expression by RstA. (**A through F**) qRT-PCR analysis of the *mrkI*, *mrkA*, *mrkB*, *mrkC*, *mrkD*, and *mrkF* mRNA. The level of *mrkI*, *mrkA*, *mrkB*, *mrkC*, *mrkD*, *and mrkF* mRNA in *ΔrstA* was significantly decreased compared to that in WT (****, *P* < 0.0001). (**G and H**) Assay of *mrkI and mrkA* promoter activity. The promoter activity of *mrkI* and *mrkA* was significantly lower in *ΔrstA* than in WT (****, *P* < 0.0001). (**I and J**) EMSA of the interaction between the promoter-proximal DNA region of the *mrkI* and *mrkA* and His-RstA protein. The promoter-proximal DNA region of *mrkI* and *mrkA* was incubated with increasing amounts of purified His-RstA protein. His-RstA protein did not bind to the *mrkI* fragment. The interaction between the promoter-proximal DNA region of the *mrkA* gene and purified His-RstA protein caused the formation of the *mrkA*-His-RstA complex, which was visualized as a DNA band with decreased mobility.

Subsequently, the regulatory effect of *rstA* on *mrkI* and *mrkABCDF* was investigated using qRT-PCR, promoter activity assays, and electrophoretic mobility shift assay (EMSA). As shown in Fig. 5A through F, qRT-PCR results indicated that *mrkI*, *mrkA*, *mrkB*, *mrkC*, *mrkD*, and *mrkF* mRNA levels were significantly lower in *ΔrstA* than in WT, suggesting that *rstA* has a positive regulatory effect on the expression of these genes. Recombinant *lacZ* plasmids containing upstream promoter regions were introduced in *ΔrstA* and WT strains to evaluate the regulatory effect of *rstA* on the promoter activity of *mrkI* and *mrkA*. As shown in Fig. 5G and H, the promoter activity (Miller units) of *mrkI* and *mrkA* in *ΔrstA* was significantly lower than in WT, indicating that *rstA* activates *mrkI* and *mrkABCDF* transcription by regulating their promoter region. EMSA was used to detect whether *rstA* directly binds to the promoter region of *mrkI* and *mrkA*. As shown in Fig. 5I and J, His-RstA did not bind to *mrkI* and the negative control 16S rRNA gene fragment (Fig. S3) but bound to the promoter region of *mrkA* in a dose-dependent manner. This indicates that *rstA* directly regulates *mrkA* by binding to its promoter but indirectly regulates *mrkI*.

## DISCUSSION

*K. pneumoniae* is a common cause of nosocomial and community-acquired infections (40). This bacterium regulates its virulence by sensing environmental stimuli, primarily through bacterial TCSs. RstBA is a TCS present in many *Enterobacteriaceae* species; however, its regulatory role in *K. pneumoniae* virulence remains largely unknown.

In this study, we investigated the effects of the TCS regulator RstA on biofilm formation and virulence in the pathogenic strain ATCC43816. Our findings show that biofilm formation, serum resistance, and virulence in *ΔrstA* were significantly reduced compared to that in WT. These results suggest that RstA positively regulates biofilm formation, serum resistance, and *in vivo* virulence in ATCC43816. Previous studies have shown that RstA regulates biofilm formation and virulence across various bacterial species. Huang L et al. found that RstA and RstB regulate the adhesion of *Vibrio alginolyticus* in response to environmental changes such as temperature, pH, and hunger. Silencing of *rstA* and *rstB* leads to impaired adhesion, biofilm production, motor ability, hemolysis, and virulence of *Vibrio alginolyticus* (23). Terceti MS et al. showed that RstAB is a major regulator of the toxicity and multiple cellular functions of *Photobacterium damselae subsp. damselae*. Mutants of *rstA* and *rstB* exhibit impaired secretion of many type II secretory system-dependent proteins, including the three significant cytotoxins Dly, PhlyP, PhlyC, and other uncharacterized proteins (24). Liu Y et al. found that RstA promotes EHEC O157 LEE gene expression, *in vitro* adhesion, and *in vivo* colonization through indirect regulation. RstA can also directly bind the promoter regions of *hdeA* and *yeaI* to enhance acid resistance and reduce biofilm formation by regulating the concentration of c-di-GMP. RstA is vital in regulating virulence, acid resistance, and biofilm formation in EHEC O157 (25). Gao et al. study found that *rstA* mutation weakened the virulence of APEC, and *rstA* affected the virulence of APEC E058 by directly regulating the expression of acid resistance gene *hdeD* (28). However, Cabeza ML et al. reported that RstA downregulates the expression of three RpoS controlling genes (*narZ*, *spvA*, and *bapA*) and inhibits biofilm formation in *Salmonella enterica* (26). Although *rstA* has 26%–45% nucleotide identity in different Enterobacteriaceae species, our results seem to differ from those of these prior studies, possibly because the function of RstA differs between the species.

To further investigate the mechanism by which RstA positively regulates biofilm formation and virulence in ATCC43816, we performed WT and *ΔrstA* transcriptome analysis. The analysis identified 105 DEGs (54 upregulated genes and 51 downregulated genes) in *ΔrstA*. KEGG enrichment analysis revealed that these DEGs were involved in various biological processes, including arginine and proline metabolism, phenylalanine metabolism, and quorum sensing. This suggested that RstA acts both as an activator and inhibitor, capable of regulating more complex pathways than expected. Further research was needed to clarify the role of RstA in these three biological processes.

Capsule is an essential virulence factor of *K. pneumoniae*. However, our previous experiments have tested the capsule production capacity and viscosity of the *ΔrstA* through quantitative uronic acid and viscosity experiments. The detections showed no significant difference between the *ΔrstA* and WT (Fig. S4 and S5). Our study suggested that RstA may indirectly regulate *mrkI* and directly regulate *mrkA* by binding to its promoter region, thereby increasing the expression of type 3 fimbriae and influencing biofilm formation and virulence in ATCC43816. The role of type 3 fimbriae in the biofilm formation and virulence of *K. pneumoniae* has been demonstrated (12, 41–43). Notably, *mrkI*, which regulates the expression of type 3 fimbriae, is adjacent to the operon of the type 3 fimbriae structural gene *mrkABCDF* in *K. pneumoniae* (13). *mrkI* is predicted to encode a LuxR-type transcriptional factor, and studies have shown that *mrkI* positively regulates the expression of type 3 fimbriae. Moreover, *mrkI* mutants exhibit a significantly decreased ability to form biofilms on both abiotic and extracellular matrix-coated surfaces (44). *mrkABCDF* is the coding gene of type 3 fimbriae, a critical virulence factor in *K. pneumoniae* that contributed to its colonization and adhesion. It is a significant medium for bacterial adhesion and a major pathogenic factor in *K. pneumoniae* (12). Deletion of *mrkA* significantly reduces adherence to abiotic surfaces and biofilm formation in continuous flow cavities, and *mrkA* expression is a significant determinant of biofilm formation (45). Ogasawara et al. found that the transcription of *csgD* in *E. coli* is repressed by the overexpression of RstA (22). CsgD controls the expression of several genes that regulate *E. coli* curli formation (46). However, our study found that RstA positively regulated the expression of type 3 fimbriae in ATCC43816, which seems to contrast with the results of the Ogasawara study. This may be due to type 3 fimbriae being uniquely expressed in *K. pneumoniae* and the different regulatory mechanisms of the RstBA TCS fimbriae across species. Since the expression levels of other genes also changed in the RNA-seq results (Table S2), RstA’s regulation of virulence may be related to other mechanisms besides type 3 fimbrial-related regulatory genes, which is worthy of further study.

In summary, this study demonstrated that RstA positively regulates the transcription of the *mrkABCDF* gene by regulating *mrkI* transcription indirectly and *mrkA* transcription directly, contributing significantly to biofilm formation and the virulence of ATCC43816. These findings offer a solid foundation for identifying new genetic targets, such as RstA, to control *K. pneumoniae* infections, which is particularly critical given the current rise in resistance to various antibacterial agents, including the increasing prevalence of carbapenem-resistant hypervirulent *K. pneumoniae*.

## Data Availability

All the data supporting this work are included in the figures or can be found in the supplemental material. The RNA-seq data in this study are available in NCBI’s Sequence Read Archive (SRA) database under BioProject ID PRJNA1227175.

## SUPPLEMENTAL MATERIAL

Fig. S1 spectrum.03076-24-s0001.eps Relative expression of rstA in WT and ΔrstA.

Fig. S2 spectrum.03076-24-s0002.tif Histopathological histograms of lung and liver in mice.

Fig. S3 spectrum.03076-24-s0003.eps EMSA of the interaction between the promoter-proximal DNA region of 16s rRNA gene and His-RstA protein.

Fig. S4 spectrum.03076-24-s0004.eps Results of quantitative uronic acid detection.

Fig. S5 spectrum.03076-24-s0005.eps Results of quantitative viscosity detection.

Table S1 spectrum.03076-24-s0006.docx Antimicrobial susceptibility profile for ATCC43816, ΔrstA, ΔrstA/cprstA, and ΔrstA/cpET28a.

Table S2 spectrum.03076-24-s0007.docx Differentially expressed genes in ΔrstA relative to the wild-type strain ATCC43816.

ASM does not own the copyrights to Supplemental Material that may be linked to, or accessed through, an article. The authors have granted ASM a non-exclusive, world-wide license to publish the Supplemental Material files. Please contact the corresponding author directly for reuse.
