# Supplementary figures and images for: Regulation of type 3 fimbria expression by RstA affects biofilm formation and virulence in Klebsiella pneumoniae ATCC43816
Source: Microbiol Spectr. 2025 May 15;13(6):e03076-24. doi: 10.1128/spectrum.03076-24 (PMC12131781; doi:10.1128/spectrum.03076-24)

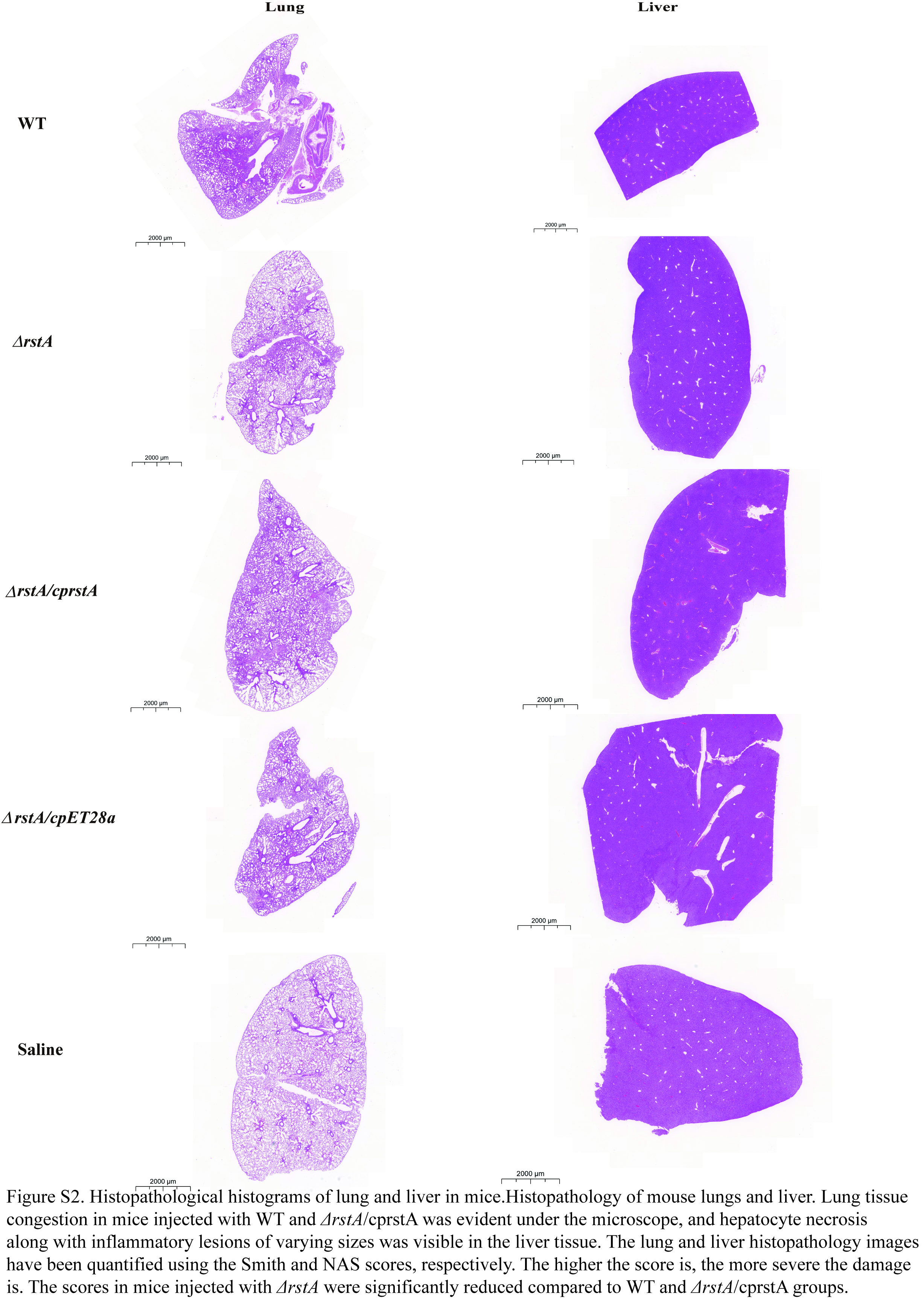

Supplement: Fig. S2 — Histopathological histograms of lung and liver in mice. [file spectrum.03076-24-s0002.tif]
