# Supplementary material for: Regulation of type 3 fimbria expression by RstA affects biofilm formation and virulence in Klebsiella pneumoniae ATCC43816
Source: Microbiol Spectr. 2025 May 15;13(6):e03076-24. doi: 10.1128/spectrum.03076-24 (PMC12131781; doi:10.1128/spectrum.03076-24)
Supplement: Table S1 — Antimicrobial susceptibility profile for ATCC43816, ΔrstA, ΔrstA/cprstA, and ΔrstA/cpET28a. [file spectrum.03076-24-s0006.docx]

| **Table S1. Antimicrobial susceptibility profile for ATCC43816, *ΔrstA*,** ***ΔrstA/cprstA* and *ΔrstA/cpET28a*.** | | | | |
| --- | --- | --- | --- | --- |
| **Antimicrobials^a, b^** | **ATCC43816**  **(μg/mL)** | ***ΔrstA* (μg/mL)** | ***ΔrstA/cprstA* (μg/mL)** | ***ΔrstA/cpET28a***  **(μg/mL)** |
| AMK | <=8 | <=8 | <=8 | <=8 |
| ATM | <=2 | <=2 | <=2 | <=2 |
| CZ | <=2 | <=2 | <=2 | <=2 |
| FOX | <=4 | <=4 | <=4 | <=4 |
| CAZ | <=1 | <=1 | <=1 | <=1 |
| CRO | <=1 | <=1 | <=1 | <=1 |
| C | <=4 | <=4 | <=4 | <=4 |
| CIP | <=0.5 | <=0.5 | <=0.5 | <=0.5 |
| COL | <=1 | <=1 | <=1 | <=1 |
| ETP | <=0.25 | <=0.25 | <=0.25 | <=0.25 |
| MI | <=2 | <=2 | <=2 | <=2 |
| LEV | <=1 | <=1 | <=1 | <=1 |
| IPM | 0.5 | 0.5 | 0.5 | 0.5 |
| MEM | <=0.125 | <=0.125 | <=0.125 | <=0.125 |
| MXF | <=0.5 | <=0.5 | <=0.5 | <=0.5 |
| TET | <=2 | <=2 | <=2 | <=2 |
| TIG | <=1 | <=1 | <=1 | <=1 |
| CFP | <=1 | <=1 | <=1 | <=1 |

a, Abbreviations: AMK, amikacin; ATM, aztreonam; CZ, cefazolin; FOX, cefoxitin; CAZ, ceftazidime; CRO, ceftriaxone; C, chloramphenicol; CIP, ciprofloxacin; COL, colistin; ETP, ertapenem; MI, Minocycline; LEV, Levofloxacin; IPM, imipenem; MEM, meropenem; MXF, moxifloxacin; TET, tetracycline; TIG, tigecycline; CFP, Cefepime.

b, The MIC values of the strains were in the same range of sensitivity, intermediation and resistance, and the difference in concentration was considered to be no difference in drug sensitivity results within two dilution multiples.
