# Supplementary material for: Regulation of type 3 fimbria expression by RstA affects biofilm formation and virulence in Klebsiella pneumoniae ATCC43816
Source: Microbiol Spectr. 2025 May 15;13(6):e03076-24. doi: 10.1128/spectrum.03076-24 (PMC12131781; doi:10.1128/spectrum.03076-24)
Supplement: Table S2 — Differentially expressed genes in ΔrstA relative to the wild-type strain ATCC43816. [file spectrum.03076-24-s0007.docx]

**Table S2 Differentially expressed genes in *ΔrstA* relative to the wild-type strain ATCC43816**

| **Gene_id** | **Gene name** | **Log_2_FC** | ***P* value** |
| --- | --- | --- | --- |
| **Downregulated expressed genes** | | | |
| VK055_RS05235 | *paaB* | -2.7715 | 0.00237 |
| VK055_RS05230 | *paaC* | -3.2966 | 1.82E-05 |
| VK055_RS05215 | *paaF* | -6.82754 | 0.000517 |
| VK055_RS22995 | VK055_RS22995 | -5.62199 | 0.040965 |
| VK055_RS13345 | *hpaI* | -2.18842 | 0.007168 |
| VK055_RS21185 | VK055_RS21185 | -5.62199 | 0.040965 |
| VK055_RS09800 | VK055_RS09800 | -3.18383 | 0.021567 |
| VK055_RS03470 | VK055_RS03470 | -5.62199 | 0.040965 |
| VK055_RS04695 | *asr* | -10.3875 | 4.62E-61 |
| VK055_RS02385 | *asr* | -9.21839 | 5.72E-52 |
| VK055_RS22910 | VK055_RS22910 | -2.03336 | 2.02E-06 |
| VK055_RS05115 | VK055_RS05115 | -2.24391 | 4.9E-06 |
| VK055_RS00835 | VK055_RS00835 | -2.0723 | 0.000334 |
| VK055_RS02060 | VK055_RS02060 | -3.18383 | 0.021567 |
| VK055_RS21275 | VK055_RS21275 | -2.40257 | 0.041032 |
| VK055_RS10040 | VK055_RS10040 | -2.40257 | 0.041032 |
| VK055_RS17140 | VK055_RS17140 | -2.22754 | 0.011803 |
| VK055_RS17445 | VK055_RS17445 | -2.46864 | 0.010034 |
| VK055_RS00955 | VK055_RS00955 | -5.62199 | 0.040965 |
| VK055_RS27890 | VK055_RS27890 | -3.76287 | 0.002134 |
| VK055_RS11450 | VK055_RS11450 | -2.28946 | 0.020952 |
| VK055_RS17455 | VK055_RS17455 | -2.52675 | 0.027043 |
| VK055_RS14495 | *ulaR* | -2.27453 | 0.001436 |
| VK055_RS20550 | VK055_RS20550 | -5.84019 | 0.022677 |
| VK055_RS08115 | VK055_RS08115 | -2.38183 | 0.014485 |
| VK055_RS22380 | VK055_RS22380 | -3.31972 | 0.013412 |
| VK055_RS26705 | VK055_RS26705 | -6.60726 | 0.001437 |
| VK055_RS15510 | *sorE* | -2.74703 | 0.011832 |
| VK055_RS03810 | VK055_RS03810 | -2.84572 | 0.007865 |
| VK055_RS08120 | VK055_RS08120 | -3.55824 | 0.005282 |
| VK055_RS21335 | VK055_RS21335 | -6.02968 | 0.012731 |
| VK055_RS04235 | VK055_RS04235 | -7.18728 | 7.55E-05 |
| VK055_RS09940 | VK055_RS09940 | -2.28946 | 0.020952 |
| VK055_RS18310 | *nikE* | -2.30107 | 0.008459 |
| VK055_RS08030 | *nfsA* | -2.55052 | 0.006966 |
| VK055_RS21825 | VK055_RS21825 | -2.40257 | 0.041032 |
| VK055_RS03900 | VK055_RS03900 | -2.08484 | 0.043991 |
| VK055_RS14245 | VK055_RS14245 | -3.94205 | 0.000885 |
| VK055_RS28220 | *pduT* | -5.84019 | 0.022677 |
| VK055_RS22220 | VK055_RS22220 | -3.15816 | 4.53E-06 |
| VK055_RS20840 | VK055_RS20840 | -3.85524 | 0.00137 |
| VK055_RS09495 | VK055_RS09495 | -5.62199 | 0.040965 |
| VK055_RS22270 | *cysD* | -2.05792 | 0.013231 |
| VK055_RS24770 | VK055_RS24770 | -5.62199 | 0.040965 |
| VK055_RS21495 | *mrkI* | -5.84019 | 0.022677 |
| VK055_RS04970 | *rstA* | -6.9643 | 2.46E-16 |
| VK055_RS12075 | *gspE* | -3.02489 | 0.003515 |
| VK055_RS25755 | VK055_RS25755 | -6.02968 | 0.012731 |
| VK055_RS25765 | VK055_RS25765 | -2.3567 | 0.000861 |
| VK055_RS00985 | *tssG* | -6.02968 | 0.012731 |
| VK055_RS04190 | VK055_RS04190 | -2.64109 | 0.017862 |
| **Upregulated expressed genes** | | | |
| VK055_RS11510 | VK055_RS11510 | 2.021715 | 0.008135 |
| VK055_RS17090 | VK055_RS17090 | 2.039101 | 0.023071 |
| VK055_RS20140 | *dhaT* | 2.408662 | 0.004369 |
| VK055_RS01710 | *dmpG* | 3.006009 | 0.034867 |
| VK055_RS17290 | VK055_RS17290 | 3.2919 | 0.013412 |
| VK055_RS21190 | VK055_RS21190 | 6.015093 | 0.012731 |
| VK055_RS02910 | VK055_RS02910 | 3.156024 | 0.021567 |
| VK055_RS23005 | *speB* | 3.2919 | 0.013412 |
| VK055_RS03530 | *hpxA* | 5.607472 | 0.040965 |
| VK055_RS01810 | VK055_RS01810 | 6.468428 | 0.002435 |
| VK055_RS24650 | *glpC* | 2.174969 | 0.000014 |
| VK055_RS10865 | VK055_RS10865 | 6.332553 | 0.004174 |
| VK055_RS22840 | VK055_RS22840 | 2.373974 | 0.041032 |
| VK055_RS05980 | VK055_RS05980 | 3.2919 | 0.013412 |
| VK055_RS18420 | VK055_RS18420 | 2.633467 | 0.000495 |
| VK055_RS05055 | VK055_RS05055 | 5.825631 | 0.022677 |
| VK055_RS28095 | VK055_RS28095 | 5.607472 | 0.040965 |
| VK055_RS03265 | VK055_RS03265 | 6.182537 | 0.007243 |
| VK055_RS10845 | VK055_RS10845 | 3.155594 | 0.001598 |
| VK055_RS03330 | VK055_RS03330 | 3.156024 | 0.021567 |
| VK055_RS22925 | VK055_RS22925 | 5.825631 | 0.022677 |
| VK055_RS11460 | VK055_RS11460 | 2.055941 | 0.043991 |
| VK055_RS21410 | VK055_RS21410 | 2.46341 | 0.00067 |
| VK055_RS13475 | *fosA* | 2.039101 | 0.023071 |
| VK055_RS14295 | *yjfF* | 2.373974 | 0.041032 |
| VK055_RS20135 | VK055_RS20135 | 3.006009 | 0.034867 |
| VK055_RS11615 | VK055_RS11615 | 6.706927 | 0.000858 |
| VK055_RS29525 | VK055_RS29525 | 2.202301 | 0.001859 |
| VK055_RS03605 | VK055_RS03605 | 6.182537 | 0.007243 |
| VK055_RS12900 | VK055_RS12900 | 3.155594 | 0.001598 |
| VK055_RS28785 | VK055_RS28785 | 6.182537 | 0.007243 |
| VK055_RS29660 | VK055_RS29660 | 2.498147 | 0.027043 |
| VK055_RS16500 | *rhaA* | 3.006009 | 0.034867 |
| VK055_RS05370 | VK055_RS05370 | 2.271978 | 0.008459 |
| VK055_RS04220 | VK055_RS04220 | 6.812856 | 0.000517 |
| VK055_RS22675 | VK055_RS22675 | 3.735007 | 0.002134 |
| VK055_RS11735 | VK055_RS11735 | 5.607472 | 0.040965 |
| VK055_RS20495 | VK055_RS20495 | 3.914169 | 0.000885 |
| VK055_RS18040 | VK055_RS18040 | 5.163131 | 5.36E-23 |
| VK055_RS01160 | VK055_RS01160 | 2.817082 | 0.007865 |
| VK055_RS14355 | VK055_RS14355 | 6.468428 | 0.002435 |
| VK055_RS17435 | VK055_RS17435 | 2.373974 | 0.041032 |
| VK055_RS22535 | VK055_RS22535 | 6.015093 | 0.012731 |
| VK055_RS27340 | VK055_RS27340 | 2.271978 | 0.008459 |
| VK055_RS00885 | VK055_RS00885 | 4.217006 | 0.000164 |
| VK055_RS25840 | *iroN* | 2.161869 | 0.030348 |
| VK055_RS12765 | *oadA* | 6.015093 | 0.012731 |
| VK055_RS18970 | *tusB* | 6.332553 | 0.004174 |
| VK055_RS11225 | *tauB* | 6.592601 | 0.001437 |
| VK055_RS00800 | *tdcC* | 3.636327 | 0.003346 |
| VK055_RS21440 | *fimB* | 2.53351 | 0.002276 |
| VK055_RS20620 | VK055_RS20620 | 6.468428 | 0.002435 |
| VK055_RS05375 | VK055_RS05375 | 2.498147 | 0.027043 |
| VK055_RS14725 | *yjeJ* | 5.825631 | 0.022677 |
